# Aerobic Exercise Training Exerts Beneficial Effects Upon Oxidative Metabolism and Non-Enzymatic Antioxidant Defense in the Liver of Leptin Deficiency Mice

**DOI:** 10.3389/fendo.2020.588502

**Published:** 2020-11-27

**Authors:** Matheus Santos de Sousa Fernandes, Lucas de Lucena de Simões e Silva, Márcia Saldanha Kubrusly, Talitta Ricarlly Lopes de Arruda Lima, Cynthia Rodrigues Muller, Anna Laura Viacava Américo, Mariana Pinheiro Fernandes, Bruno Cogliati, José Tadeu Stefano, Claudia Jacques Lagranha, Fabiana S. Evangelista, Claudia P. Oliveira

**Affiliations:** ^1^Laboratório de Gastroenterologia Clínica e Experimental LIM-07, Division of Clinical Gastroenterology and Hepatology, Hospital das Clínicas HCFMUSP, Department of Gastroenterology, Faculdade de Medicina, Universidade de São Paulo, São Paulo, Brazil; ^2^Laboratório de Transplante e Cirurgia do Fígado (LIM-37), Hospital das Clínicas HCFMUSP, Faculdade de Medicina, Universidade de São Paulo, São Paulo, Brazil; ^3^Federal University of Pernambuco, Academic Center of Vitoria, Pernambuco, Brazil; ^4^Department of Experimental Pathophysiology, Faculdade de Medicina, Universidade de São Paulo, São Paulo, Brazil; ^5^Department of Pathology, School of Veterinary Medicine and Animal Science, University of São Paulo, São Paulo, Brazil; ^6^School of Arts, Science and Humanities, University of São Paulo, São Paulo, Brazil

**Keywords:** liver disease, oxidative metabolism, antioxidant defense, physical exercise, leptin deficiency

## Abstract

Non-alcoholic fatty liver disease (NAFLD) is one of the most common forms of liver disease, which is associated with several etiological factors, including stress and dysfunction in oxidative metabolism. However, studies showed that aerobic exercise training (AET) can combat the oxidative stress (OS) and improves mitochondrial functionality in the NAFLD. To test the hypothesis that AET improves oxidative metabolism and antioxidant defense in the liver of *ob/ob* mice. Male *ob/ob* mice with eight weeks old were separated into two groups: the sedentary group (S), n=7, and the trained group (T), n=7. The T mice were submitted to an 8-week protocol of AET at 60% of the maximum velocity achieved in the running capacity test. Before AET, no difference was observed in running test between the groups (S=10.4 ± 0.7 min *vs.* T= 13 ± 0.47 min). However, after AET, the running capacity was increased in the T group (12.8 ± 0.87 min) compared to the S group (7.2 ± 0.63 min). In skeletal muscle, the T group (26.91 ± 1.12 U/mg of protein) showed higher citrate synthase activity compared with the S group (19.28 ± 0.88 U/mg of protein) (p =0.006). In the analysis of BW evolution, significant reductions were seen in the T group as of the fourth week when compared to the S group. In addition, food intake was not significant different between the groups. Significant increases were observed in the activity of enzymes citrate synthase (p=0.004) and β-HAD (p=0.01) as well as in *PGC-1α* gene expression (p=0.002) in the liver of T group. The levels of TBARs and carbonyls, as well as SOD, CAT and GST were not different between the groups. However, in the nonenzymatic antioxidant system, we found that the T group had higher sulfhydryl (p = 0.02), GSH (p=0.001) and GSH/GSSG (p=0.02) activity. In conclusion, the AET improved body weight evolution and the aerobic capacity, increased the response of oxidative metabolism markers in the liver such as *PGC-1α* gene expression and citrate synthase and β-HAD enzyme activities in *ob/ob* mice. In addition, AET improved the non-enzymatic antioxidant defense and did not change the enzymatic defense.

## Introduction

Non-alcoholic fatty liver disease (NAFLD) is characterized by the accumulation of intrahepatic lipids, reaching an absolute level of 5% of the total hepatic content (1, 2). This pathological process is associated with etiologic factors and metabolic comorbidities, such as insulin resistance (IR), cardiovascular diseases (CVD), and type 2 diabetes (T2DM), and is responsible for the progression of clinical staging for non-alcoholic steatohepatitis (NASH), liver cirrhosis and hepatocellular carcinoma (HCC) (3). Currently, NAFLD is one of the most common causes of liver diseases and affects approximately 25%–30% of people around the world. In Western society, its prevalence reaches alarming levels of 20%–30% in overweight adults and 90% in obese adults (3). In addition, the process of industrialization, an imbalance in the intake of macronutrients and sedentarism contribute to the development and progression of this pathology (4).

The accumulation of intrahepatic lipids in NAFLD may be due to the lipid metabolism imbalance characterized by a reduction in oxidative capacity and/or an increase in lipogenic activity. In addition, metabolic imbalance provides functional overload in the mitochondria, which ultimately causes mitophagy, the process of mitochondrial degradation, and the subsequent autolysis of this organelle (5). Part of the processes involved in the hepatic metabolic response is regulated by the expression of peroxisome proliferator-activated receptor gamma coactivator 1-alpha (PGC1-α) and its signaling pathway and have important repercussions for lipid and glucose metabolism control, mainly in the stabilization of energy deficits in the liver and in the whole organism that are triggered by overweight and obese phenotypes and NAFLD (6).

One of the important factors that contributes to the progression of the NAFLD spectrum and the progression to advanced disease is the excessive production of reactive oxygen species (ROS) (mainly superoxide anions and peroxides), which are responsible for the direct stimulation of the chronic inflammatory process, the deregulation of oxidative metabolism, and the promotion of fibrogenesis through the activation of hepatic stellate cells followed by the increased expression of type I collagen in the hepatic parenchyma (7, 8). Among the substances that are produced is hydrogen peroxide (H_2_O_2_), which causes the activation of macrophages and consequent production of pro-inflammatory cytokines in the intracellular environment.

Regarding pharmacological treatment, there is no FDA-approved direct therapy for NAFLD, although some medications are used to control etiological factors such as hyperinsulinemia and IR (3, 9). Nevertheless, changes in lifestyle, including adherence to regular aerobic exercise training (AET) and better dietary patterns, are recommended as the first line of treatment for NAFLD (10, 11). It has been demonstrated that AET exerts an important role in the control of NAFLD physiopathology by reducing body weight and oxidative stress, improving oxidative metabolism, and reducing steatosis, inflammation and the levels of fibrosis biomarkers, such as cytokeratin 18 (12–14). In addition, AET at a moderate-vigorous intensity increases cardiorespiratory capacity and prevents the development and progression of NAFLD (15). Moreover, studies have shown that AET may decrease the detrimental effect of ROS production in addition to increasing the rate of muscle contraction, beta-oxidation and mitochondrial biogenesis (16–18).

To better understand the role of AET for the prevention and treatment of NAFLD, our group previous studied the effect of AET in the liver of *ob/ob* mice. To our surprise, despite the body weight gain and physical exercise tolerance were improved in trained ob/ob mice, the AET failed to prevent NAFLD (19). The results of this study do not allow to exclude other effects of AET that could offer benefits to the liver and improve the progression of the disease. Thus, considering that the liver’s metabolic condition is a key point in the development and progression of NASH, and that there is still a knowledge gap about the role of APT on hepatic oxidative metabolism and antioxidant defense in *ob/ob* mice, the aim of the present study was to test the hypothesis that AET improves oxidative metabolism and antioxidant defense in the liver of *ob/ob* mice.

## Materials and Methods

### Animals

Male 8-week-old C57BL/6 *ob/ob* mice [From the Laboratório de Gastroenterologia Clínica e Experimental (LIM-07)], matched for body weight, were separated randomly into two groups: sedentary (S, n=7) and trained (T, n=7). The mice were housed in a temperature-controlled environment (22 ± 2°C) with a 12-h light/12-h dark cycle and free access to tap water and food (Nuvilab - Nuvital Nutrientes S/A, Brazil). The procedures were performed according to the recommendations guidelines of the Animal Experimentation Service of the Medical School of the University of São Paulo and were approved by the Ethics Committee on the Use of Animals (CEUA) of the School of Medicine of University of Sao Paulo (Number 040/17).

### Running Test

The test was performed before, in the fourth and eighth weeks of AET using a progressive method without inclination described by Ferreira et al. (20) in both groups (S; T). The protocol started with a speed of 0.4 km/h and has been increased by 0.2 km/h every 3 min until mice exhaustion, which was characterized by the impossibility of maintaining the standard rate.

### Aerobic Exercise Training

T mice were trained during the dark cycle on a motorized treadmill (Inbramed KT 10200, Porto Alegre, Brazil) for 1 h/day at 60% of maximal velocity, five times per week for eight weeks. The AET intensity progressively increased; it started at 0.3 km/h and was adjusted after the running capacity test performed in the fourth week. Sedentary mice were placed on the treadmill for 10 min twice weekly at 0.2 km/h to minimize treadmill stress.

### Body Weight and Food Intake

Body weight was measured weekly at the same time of day using a digital balance (Gehaka, Model BK4001, São Paulo, Brazil), and 24-h food intake was determined weekly throughout the study. The mice were housed in cages containing 3-4 mice.

### Death Procedure

Forty-eight hours after the end of the last training session, the mice were anesthetized with an intraperitoneal ketamine hydrochloride (0.5 ml/kg), exsanguination was performed, and liver and muscle tissues were removal. The skeletal muscle was removed for determination of citrate synthase activity. Next, the liver was harvested, weighed, and processed according to the experiments described below.

### Gene Expression

After liver and skeletal muscle tissue (50 mg) pulverization at liquid nitrogen temperatures, total RNA was prepared using Trizol^®^ (Invitrogen Life Technologies, Carlsbad, CA, USA) according to the manufacturer’s recommendations (8). Total RNA was dissolved in RNase-free water, and the RNA concentration was determined by spectrophotometry. RNA purification was determined based on a 260/280 nm ratio >1.8. Samples were kept at −80°C until processing by reverse transcription quantitative polymerase chain reaction (RT-qPCR) analysis.

After extracting the total RNA, the expression levels of multiple genes in the liver were measured. The genes that were measured and the primers used to measure them are as follows: *sterol regulatory element-binding protein 1 (SREBP1*) *(5´*GCG CTA CCG GTC TTC TAT CA*; 3´* GGA TGT AGT CGA TGG CCT TG); peroxisome proliferator-activated receptor alpha (*PPAR-α)* (5´ ATG CCA GTA CTG CCG TTT TC; 3´ TTG CCC AGA GAT TTG AGG TC); 3´ TCA AAC AGT TCC ACC TGC TG); peroxisome proliferator-activated receptor gamma coactivator 1-alpha (*PGC-1α)* (5´CTA CAG ACA CCG CAC ACA TCGC; 3´ GGA TGT AGT CGA TGG CCT TG); and endogenous control gene *β-actin* (5´ TGT TAC CAA CTG GGA CGA CA; 3´ GGG GTG TTG AAG GTC TCA AA). The levels were analyzed in the liver with the polymerase Rotor gene 3000 (Corbett Research, Sydney, Australia) using the Superscript™ III Platinum^®^ One-Step Quantitative RT-PCR System (Invitro-gen Life Technologies, Carlsbad, USA) according to the instructions provided by the manufacturer. Reactions lacking reverse transcriptase were also run to generate controls for the assessment of genomic DNA contamination. Fluorescence changes were monitored after each cycle (72°C, ramping to 99°C at 0.2°C/s, with continuous fluorescence readings), and melting curve analyses were performed at the end of the cycles to verify the PCR product identity. After the experiment was performed, the relative amount of each intergroup gene was calculated by the ^ΔΔ^Ct coefficient, as provided by the device software (21).

### Enzyme Activity

Citrate synthase is the first enzyme in the Krebs cycle and is particularly important for the catalysis and condensation of acetyl CoA with oxaloacetate for the formation of citrate, the first product of the Krebs cycle. Moreover, this enzyme is an indicator of trainability, as described by Le Page et al. (22). Briefly, the reaction was carried out in a mixture containing Tris-HCl (pH = 8.2), magnesium chloride (MgCl), ethylenediamine-tetra-acetic acid (EDTA), 0.2–5.5 dithiobis (2-nitrobenzoic acid) (E = 13.6 μmol/(ml.cm), 3 acetyl CoA, 5 oxaloacetate and 0.3 mg/ml of homogenized hepatic and skeletal muscle tissue. The enzymatic activity was evaluated by measuring the change in the absorbance rate at 412 nm for 3 min at a temperature of 25°C. Citrate levels are expressed as U/mg of protein (23).

For the measurement of 3-hydroxyacyl-CoA dehydrogenase (β-HAD) activity in liver, the liver was homogenized in a solution containing the following (in millimolar concentrations): 20 Tris-HCl (pH 7.4 at 4°C), 50 NaCl, 50 NaF, 5 Sodium pyrophosphate, 0.25 sucrose, and dithiothreitol, with protease inhibitor cocktail (Sigma) and phosphatase inhibitor cocktail (Sigma) (24). After homogenization, the protein contents of the homogenates were determined by the Bradford protein assay. For β-HAD activity, 15 µg of protein was incubated in a reaction mixture containing the following (in millimolar concentrations): 50 imidazole (pH 7.4), 0.15 NADH, and 0.1 acetoacetyl-CoA (omitted for the control). β-HAD activity was determined at 340 nm by measuring the consumption of NADH (ϵ 6.22 µmol·ml^-1^·cm^-1^) over 5 min (in 30-s intervals), β-HAD levels are expressed as U/mg of protein. The procedures that were used were described previously by Ito et al. (25).

### Oxidative Stress

In the present study, lipid peroxidation was also evaluated through substances reactive to thiobarbituric dosage. In this assay 300 μg of protein were mixed to 30% (w/v) trichloroacetic acid (TCA) and 3 mM TRIS buffer (pH 7.4) in equal volumes and stirred. This mixture was centrifuged at 2,500 g for 10 min, the supernatant was mixed with 0.73% thiobarbituric acid and boiled at 100°C for 15 min. The pink pigment yielded was measured spectrophotometrically at 535 nm at room temperature. The results were expressed as μmol of malondialdehyde (MDA/mg protein) (26, 27).

The protein oxidation was evaluated as described by Levine et al. (28). Liver samples with 300 mg of protein, 30% (w/v) TCA was added to the sample and then centrifuged for 15 min at 664 g. The pellet was resuspended in 10 mM 2,4- dinitrophenylhydrazine (DNPH) and immediately incubated in a dark room for 1 h with shaking every 15 min. The samples were washed and centrifuged three times in ethyl acetate buffer and at the end of the procedures the pellet was resuspended in 6M guanidine hydrochloride incubated for 30 min at 37 o163 C and the absorbance read at 370nm. The results were expressed as μM/mg protein (24).

### Antioxidant Defense

Superoxide dismutase (SOD) activity was determined in agreement with Misra and Fridovich (29). In this way, 300 μg of protein was used with addition of 100mM of carbonate buffer with 5mM EDTA (pH 10.2). The reaction was initiated with the addition of 150 mM epinephrine and the SOD activity was determined by the inhibition of epinephrine auto-oxidation at 30°C. The decrease in absorbance was monitored for 2 min at 480 nm and the results express in U/mg protein. Catalase activity assay has been previously described Aebi (30). Liver homogenate (300 μg of the protein) was used, with the addition of 50 mM of the phosphate buffer (pH 7.0) and 0.3 M of hydrogen peroxide and its oxidation. All enzymatic kinetics was monitored at 240 nm for 3 min at 20°C, and the results expressed as U/mg protein (27).

The activity of GST was previously described by Habig et al. (31) that evaluated as follows: 200 μg protein was added to 100 mM phosphate buffer (pH 6.5) containing 1 mM EDTA. For this solution, 1 mM reduced glutathione and 1 mM 1- chloro-4,4-dinitrobenzene (CDNB) were added so that the reaction could start. The absorbance standard for this component was monitored at 340 nm for 1 min to detect the formation of 2,4-dinitrophenol-S-glutathione (DNP-SG). One enzyme unit conjugates 10 nmol of CDNB with GSH per minute. The results were expressed as U/mg protein.

To measure the REDOX state, we measured both reduced and oxidized glutathione levels. The levels of reduced glutathione (GSH) were evaluated by adding 100 mM phosphate buffer (pH 8.0) with 5 mM EDTA to the samples (0.300 mg protein), followed by a period of 15 min incubation with O-phthalaldehyde (OPT) (1 μm) at RT. Fluorescence intensity was measured at 350 nm (excitation) and 420 nm (emission) and compared with a standard GSH curve (0.5–100 μM). The oxidized glutathione (GSSG) levels were evaluated by incubation of samples with 40 mM Nethylmaleimide for a period of 30 min in RT followed by addition of 100 mM NaOH buffer. Afterwards, the same steps of the GSH assay were followed 196 to determine the GSSG levels. The REDOX state was determined by the ratio of GSH/GSSH (32).

The measurement of total thiol groups consisted of a cold extraction buffer (50 mM Tris base, pH 7.4; 1 mM EDTA; 2 mM PMSF, 10 mM sodium orthovanadate) added to the samples, followed by incubation with 10 mM 5,5′-dithiobis (2 nitrobenzoic acid) (DTNB) at RT under a dark cover for a period of 30 min. The samples were measured at 412 nm as described by Ellman (33).

### Statistical Analysis

Data normality test was performed using the D’Agostino and Pearson test with Gaussian adjustment and were reported as the mean ± SEM. Differences between the two groups were analyzed using Unpaired Student’s t-test, except for body weight evolution, which was analyzed using one-way ANOVA for repeated measures. The Bonferroni *post hoc* test was used to determine differences between the means when a significant change was determined by ANOVA. A *p* value of less than 0.05 was statistically significant, and *Prism V6* was used.

## Results

Before AET, no difference was observed in the running capacity between the groups (S= 10.4 ± 0.7 min *vs.* T= 13 ± 0.47 min). However, after 8 weeks of AET, the running capacity was higher in the T group (12.8 ± 0.87 min) compared to the S group (7.2 ± 0.63 min). We analyzed the activity of citrate synthase in skeletal muscle as an indicator of AET efficiency. Our data demonstrated that the T group (26.91 ± 1.12 U/mg of protein) exhibited higher citrate synthase activity compared to that exhibited by the S mice (19.28 ± 0.88 U/mg of protein) (p =0.0006).

**Figure 1:**
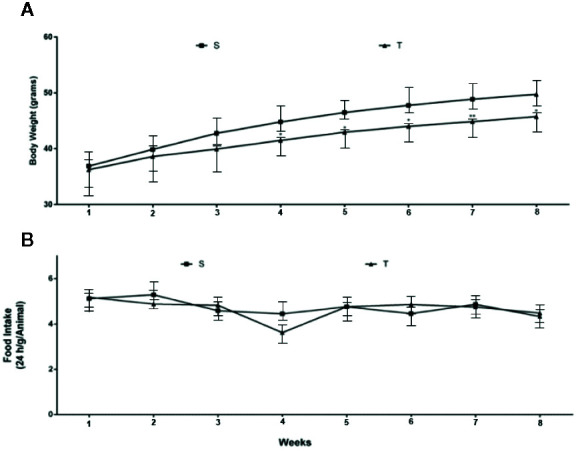
Body weight **(A)** and food intake **(B)** evaluation in *ob/ob* mice. S (n = 7) and T (n = 7).

The body weight (BW) of the animals in both groups at the beginning of AET was evaluated, and no statistically significant differences were observed (**Figure 1A**). During the experimental protocol, the T mice exhibited lower body weight beginning in the fourth week of AET compared to that exhibited by the S group (**Figure 1A**). We also measured food intake during the experimental protocol, and as showed in **Figure 1B**, no significant differences were observed between groups.

**Figure 2:**
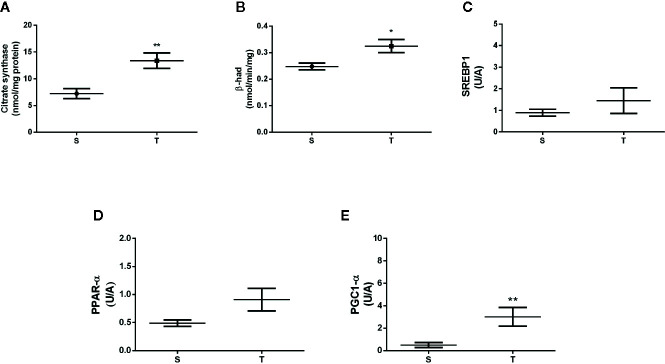
Citrate synthase activity **(A)**, β-hydroxyacetyl-codehydrogenase (β-HAD) activity **(B)** and the gene expression of *sterol regulatory element-binding protein I* (SREBP1) **(C)**, peroxisome proliferator-activated receptor alpha (PPAR-α) **(D)**, and peroxisome proliferator-activated receptor gamma coactivator 1-alpha (PGC-1α) **(E)** in the liver of *ob/ob* mice. *p < 0.05; **p < 0.01. S (n = 6) and T (n = 7).

In the liver, the T mice exhibited higher citrate synthase and β-HAD enzyme activities when compared with S mice (p=0.01 and 0.003, respectively) (**Figures 2A, B**). No differences were found in the mRNA levels of SREBP1 and PPAR-α (p=0.309 and p=0.615, respectively) (**Figures 2C, D**). In contrast, we observed a significant increase in PGC-1α mRNA expression in the T mice (p=0.002) (**Figure 2E**).

**Figure 3:**
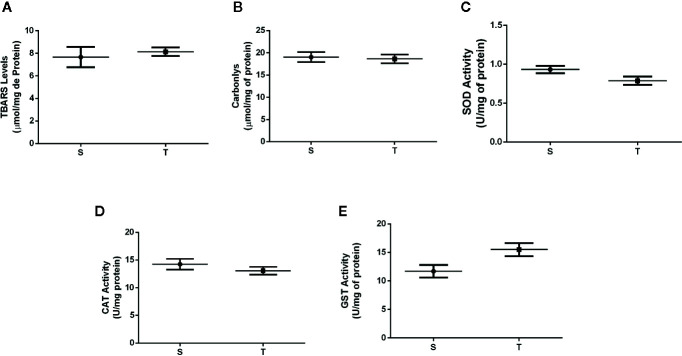
TBARS **(A)** and carbonyl levels **(B)**, enzymatic activity of superoxide dismutase (SOD) **(C)**, catalase (CAT) **(D)** and glutathione S transferase (GST) **(E)** in the liver of *ob/ob* mice. S (n = 6) and T (n = 7).

**Figure 4:**
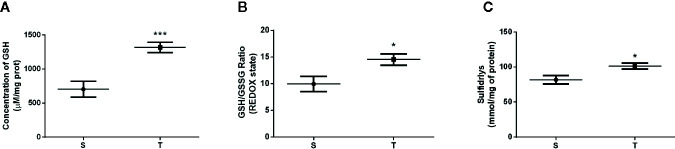
Reduced glutathione levels (GSH) **(A)**, GSH/GSSG ratio **(B)** and total levels of sulfhydryls **(C)** evaluated in the liver of *ob/ob* mice. *p < 0.05; ***p < 0.001. S (n = 6) and T (n = 7).

Regarding oxidative stress, no difference was observed in lipid and protein peroxidation between the groups (p=0.6167 and p=0.0887) (**Figures 3A, B**). The activity of antioxidant enzymes (SOD, CAT and GST) in the liver of the T and S mice were also not significantly different (p=0.06, 0.11 and p=0.08, respectively) (**Figures 3C–E**). However, the T group showed higher levels of nonenzymatic defense [GSH levels, redox state (GSH/GSSG ratio), and the amount of sulfhydryl groups] compared to those in the S group (p=0.001, p=0.02 and p=0.02) (**Figures 4A–C**, respectively).

## Discussion

Our results revealed that AET was responsible for an improvement in body weight control, stimulate oxidative metabolism and non-enzymatic antioxidant activity associated with an increase in the expression of gene related to mitochondrial biogenesis in the liver of *ob/ob* mice with NAFLD. In the present study, variables related to AET were analyzed to determine trainability that means the adaptability of responses related to physical exercise. From this perspective, the T mice showed higher mean values of running capacity (measured as the length of time spent running in minutes) compared to those of the S mice. This result corroborates our previous study, which demonstrated greater differences in trained animals compared with sedentary mice after eight weeks of AET. Additionally, we observed higher citrate synthase activity in skeletal muscle in the T group, which confirms that AET can provide higher levels of oxidative metabolism when executed in a moderate intensity.

We analyzed the evolution of BW during the eight weeks of AET and significant differences were found between the groups after the fourth week of AET. However, no differences were observed when analyzing the evolution of food intake. Similarly, in a study carried out by our group, T mice showed better control of body weight evolution, however the differences between the groups began in the third week of AET. Recently, we also published that AET at moderate intensity (60% of running capacity) during 8 weeks was capable of promoting changes in body weight in the same animal model (34). These data confirm the efficacy and reproducibility of AET in body weight control in this animal model.

It is known that the generation of NAFLD is regulated by factors including the abnormal accumulation of intrahepatic lipids, insulin resistance, and a reduction in β-oxidation capacity, but none of these factors are as important as mitochondrial dysfunction (35). Thus, we performed analysis of citrate synthase and β-HAD activities in the liver since these elements are linked to mitochondrial function. The T animals demonstrated a significant increase in both metabolic enzymes compared with the S group, which reveal the AET efficiency to improve metabolic parameters associated with hepatic mitochondrial bioenergetics.

Regarding energetic regulation, we evaluated PGC-1α gene expression in the liver of ob/ob mice and demonstrated a significant increase in the T group compared with the S group. Consistent with our findings, Gonçalves et al. (36) showed that aerobic physical activity performed on a running wheel was able to increase the level of PGC-1α gene expression, which is a key factor in the regulation of lipid metabolism, body weight and can generate benefits for important features of NAFLD. The authors clarify that the upregulation of energy functionality markers such as PGC-1α represents an adaptive response linked to aerobic exercise, which in turn can stimulate the mitochondrial renewal used to combat more severe forms of NAFLD including NASH. Thyfault et al. (37) reported that energetic dysregulations are mediated by different levels of PGC-1α gene expression. This coactivator regulates the activation and deactivation of a signaling pathway for mitochondrial biogenesis which involves PPAR- α, mitochondrial transcription factor A (TFAM), nuclear respiratory factor-1 and 2 (NFR-1 and 2). Another recent study showed that physically inactive mice have lower levels of PGC-1α gene expression and that this has repercussions for the establishment of an obese phenotype, histological damage, oxidative stress and NAFLD (18, 38, 39).

The levels of PPAR-α, despite a trend toward higher mean values in the T mice, were not significant different. Also, we observed that AET was not able to improve marker of lipogenesis in this model, since no significant difference was observed in the expression of SREBP1 in the liver. This result was also observed in the previous study by Evangelista et al. (19) suggesting that AET can improve hepatic energetic metabolism without change in lipogenesis.

In the investigation of the redox balance, we firstly found no significant differences in the levels of lipid peroxidation (TBARS) and protein oxidation (carbonyl assay) markers after the AET. Differently, Sun et al. demonstrated that 4 weeks of AET at a high intensity and for a short duration were able to increase the production of TBARs and ROS in the rat liver, whereas trained rats that received supplements of b-complex vitamins and creatine exhibited greater activity of mitochondrial complexes I, IV and V. Differences in the exercise protocol structure including type (aerobic or strength exercise), effort duration (seconds, minutes or hours), intensity (low, moderate or high), and volume associated with exposure to different diets can explain distinct results between ours and the other study.

In our study there was no difference in antioxidant defense according to the results of SOD, CAT and GST in the T group. De Sousa et al. demonstrated that AET increased SOD, CAT and GST activity in the liver and skeletal muscle in mice (40). Also Jinho Ko, Kijin Kim, 2013 demonstrated the effectiveness of an aerobic exercise protocol on a treadmill lasting 35–65 min per day, during 5 days per week in the gene expression of manganese containing superoxide dismutase (MnSOD) in the white adipose tissue of mice with obesity induced by high-fat diet (41). Again, it is possible that differences in the results are associated with the animal model since we used genetically modified mice with leptin deficiency. Furthermore, the duration of AET intervention may not have been long enough to promote these adaptations in the antioxidant enzymes.

Reducing oxidative stress is fundamental for improving not only the progression of NAFLD but also obesity and IR (10, 15, 42). Due to the results we found related to enzymatic defense, we decided to analyze some molecules related to nonenzymatic defense such as reduced and oxidized glutathione levels and the total amount of thiol groups. The functioning of this non-enzymatic antioxidant pathway is mainly mediated by tissue levels of reduced glutathione and the action of the enzyme glutathione reductase (GSH reductase). This enzyme is associated with the plasma membrane allowing the conversion of GSSG to GSH through the oxidation of electron carriers including nicotinamide adenine dinucleotide phosphate in its oxidized and reduced form (NADP^+^ and NADPH). These reactions are essential for attenuation of lipid peroxidation, protein oxidation and damage to macromolecules of cellular components, participating in the removal of reactive oxidant species with potential antioxidant activity (43, 44). Our data showed that the T animals presented significantly higher levels of all the components of nonenzymatic defense compared to those exhibited by the S mice. The increase in the redox levels in response to AET may be effective in reducing the intracellular amount of peroxide and hydroxyl peroxide, which can decrease the proinflammatory response, fibrosis and cellular apoptosis commonly found in NAFLD/NASH (32, 45). These results are of paramount importance in the spectrum of NAFLD since the activation of the action-dependent pathway of the redox state complex guarantees the liver’s protective effect against the production of bioactive compounds related to oxidative stress (45).

In conclusion, the AET improved body weight evolution and the aerobic capacity, increased the response of oxidative metabolism markers in the liver such as PGC-1α gene expression and citrate synthase and β-HAD enzyme activities in *ob/ob* mice. In addition, AET improved the non-enzymatic antioxidant defense and did not change the enzymatic defense.

## Data Availability Statement

The raw data supporting the conclusions of this article will be made available by the authors, without undue reservation.

## Ethics Statements

The animal study was reviewed and approved by the Ethics Committee on the Use of Animals (CEUA) of the School of Medicine of University of Sao Paulo (Number 040/17). No potentially identifiable human images or data are presented in this study.

## Author Contributions

MF, CP, LL, ST, and FE conceived the study idea and design. MF, LL, and FE formulated the aerobic exercise training intervention. MF, CP, LL, SJ and FE conducted animal model selection and care. MF, CP, LL, ST, CM, AV, and FE conducted interventions. MF, LL, and MK performed DNA extraction and RT-PCR analysis. MF, LS, TL, CL, MP performed the oxidative status analysis, and BC performed the histological liver analysis. MF, CM, LL, ST, and FE wrote the manuscript with review, editing, and final approval from all authors. All authors contributed to the article and approved the submitted version.

## Funding

Financial support was provided by the Coordenação de Aperfeiçoamento de Pessoal de Nível Superior (CAPES) and FACEPE (Grant number: APQ-0164-4.05/1). This study was supported by the Coordenação de Aperfeiçoamento de Pessoal de Nível Superior (CAPES) and FACEPE. We thank the Federal University of Pernambuco for their collaboration in this work.

## Conflict of Interest

The authors declare that the research was conducted in the absence of any commercial or financial relationships that could be construed as a potential conflict of interest.

## Abbreviations

AET, Aerobic exercise training; CAT, Catalase; GSH, Reduced glutathione levels; GSSG, Oxidated glutathione levels; GST, Glutathione S Transferase; NAFLD, Nonalcoholic fatty liver disease; ROS, Reactive oxygen species; SED, Sedentary Group; SOD, Superoxide Dismutase; T, Trained Group; TBARS, Thiobarbituric acid reactive species; T2DM: Type 2 diabetes mellitus.
